# Meta-Research: Positive genetic interactions have greater scientific impact but are under-represented in the literature

**DOI:** 10.1101/2025.06.25.661621

**Published:** 2025-10-27

**Authors:** Mengyi Sun, David M. McCandlish

**Affiliations:** 1 Simons Center for Quantitative Biology, Cold Spring Harbor Laboratory, Cold Spring Harbor, NY, 11724

## Abstract

Genetic interactions, where the combined effect of perturbing two genes leads to a phenotype that deviates from the expectation based on the effects of the individual perturbations, often provide important information about the functional architecture of biological systems. These interactions are commonly classified as negative or positive based on whether the phenotype of the double mutant is less than or greater than what would be expected based on the single mutant effects. When the trait in question is fitness, systematic studies of pairwise deletions have shown that negative interactions typically link genes with similar functional annotations, while positive interactions typically link genes that are less obviously related and thus often viewed as less informative. However, research in the sociology of science suggests that transformative discoveries often arise from unexpected, cross-domain linkages. To evaluate these competing perspectives, we integrated large-scale genetic interaction data in yeast with literature annotations from the Saccharomyces Genome Database and citation data from iCite. We found that positive genetic interactions are associated with greater scientific impact, as measured by citation metrics, contrary to prevailing assumptions. Nevertheless, despite their greater impact, positive interactions are significantly underrepresented in the scientific literature, suggesting they are frequently overlooked. These findings reveal the underappreciated value of positive genetic interactions and demonstrate the potential of applying sociology-of-science insights to foster biological innovation.

## Introduction

Genetic interaction refers to the phenomenon where the combined effect of disrupting two genes on a trait deviates from the expectation based on perturbing each gene individually^1^. This phenomenon has proved valuable for uncovering functional relationships among genes and pathways^2^, and has implications for a range of biological questions—from the origin of species in evolutionary biology^3^ to the genetic basis of human diseases^4^ and drug interactions^5^.

In model organisms like yeast, genetic interactions affecting fitness can be quantified as $I=f_{ab}-f_{a}\times f_{b}$ where $f_{a}$ and $f_{b}$ denote the relative fitness of single mutants and $f_{ab}$ that of the double mutant^2^. Interactions are typically classified by the sign of I: negative when I<0, positive when I>0. Prior studies have shown that negative interactions often link genes with high functional relatedness based on previous knowledge, and are therefore regarded as more functionally informative^6^. Positive interactions, which tend to link genes without apparent functional relationship, are often deemed less informative^6^. This perspective implies that studying gene pairs involved in negative interactions should be scientifically more valuable.

However, a key insight from the sociology of science is that impactful innovation often stems from unexpected linkages across conceptual domains^7^. From this perspective, positive genetic interactions, by linking genes whose functional roles are less obviously related, may offer greater potential for scientific innovation.

To evaluate whether positive or negative interactions are more scientifically impactful, we analyzed large-scale genetic interaction data from yeast, linking gene pairs to their associated scientific output using yeast gene literature^8^ and iCite citation databases^9^. Our results show that positive interactions are associated with greater scientific impact. Nevertheless, despite their empirical importance, positive interactions remain significantly underrepresented in the scientific literature. These findings challenge conventional views about functional informativeness and highlight the value of applying sociology-of-science insights to biological discovery.

## Results

### Positive genetic interactions are associated with higher scientific impact

We investigated the scientific impact of positive versus negative gene interactions by analyzing publications from the Saccharomyces Genome Database (SGD)^8^. This database links 418,111 papers to their associated genes, from which we selected the subset of publications associated with exactly two genes. We then further selected the subset of these papers where the gene pair studied exhibited a significant genetic interaction score (p < 0.05) from the large-scale screen conducted by Costanzo et al. ^2^. To assess scientific impact, we used iCite, a bibliometric database accessed via PubMed IDs provided by SGD. Our primary metric was the year-specific percentile citation rank, which compares a paper’s citation count to those of other papers published in the same year within the SGD corpus. This approach standardizes citation impact by controlling for publication year—a standard practice in scientometrics—to avoid biasing results in favor of older papers that have had more time to accumulate citations^10^.

**Figure 1:**
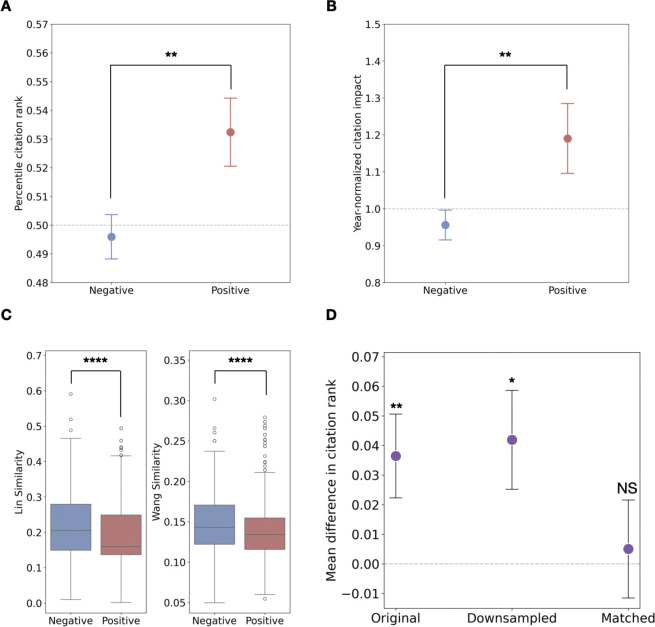
Positive interactions are linked to higher scientific impact and novelty. **(A)** Mean year-specific citation rank and **(B)** year-normalized citation impact for literature on positive (red) vs. negative (blue) interactions. **(C)** Boxplot of GO similarity measured by Lin’s method (left) and Wang’s method (right) between gene pairs involved in positive (red) and negative (blue). **(D)** Mean difference in citation rank (positive – negative) across original, downsampled, and GO-similarities-matched samples; citation advantage of positive interactions vanishes only after matching for GO-similarities. All error bars represent standard errors. Significance levels are indicated as follows: NS, *P* ≥ 0.05; *P < 0.05; **P < 0.01, ***P < 0.001; ****P<0.0001.

As shown in Figure 1A, publications focusing on positive genetic interactions are significantly more impactful than those involving negative interactions (p<0.01, t-test; p<0.01, Mann-Whitney U test), as measured by year-specific percentile citation rank. This finding is further supported by the year-normalized citation impact^11^ (a paper’s citation count divided by the average for papers published in the same year), where Figure 1B shows that studies on positive interactions tend to have ~20% more citations on average than those on negative interactions of the same age (p<0.01, t-test; p<0.01, Mann-Whitney U test). These results consistently indicate that when gene pairs involved in positive interactions are studied together, they tend to generate more impactful research.

We further explored whether positive interactions’ higher impact stems from their novelty, i.e. their propensity to link apparently disparate biological processes. Figure 1C confirms positive interaction pairs consistently exhibit lower GO similarity than negative interactions (using Lin’s^12^ and Wang’s^13^ metrics, all p<0.0001, Mann-Whitney U test), indicating greater novelty. To confirm novelty as the mediator, we analyzed citation rank differences across sample types (Figure 1D). The full dataset shows a significant citation advantage for positive interactions (p<0.01,t-test, Figure 1D, left). Crucially, when we match positive and negative interaction pairs based on their GO similarities (where lower similarity reflects greater novelty; see Methods), this advantage becomes statistically indistinguishable from zero (p=0.8,t-test, Figure 1D, right). This indicates novelty drives the observed citation advantage. To confirm that the loss of statistical significance was not a sample size artifact, we also performed random downsampling to equalize positive and negative interaction paper counts. In this downsampled scenario, the citation advantage for positive interactions remained significantly higher than zero (p<0.05, t-test, Figure 1D, middle), similar to the full sample.

Together, these findings suggest that gene pairs involved in positive genetic interactions are associated with higher-impact publications because positive genetic interactions often uncover novel connections between seemingly unrelated biological processes.

### Positive genetic interactions are underexplored in the scientific literature

**Figure 2:**
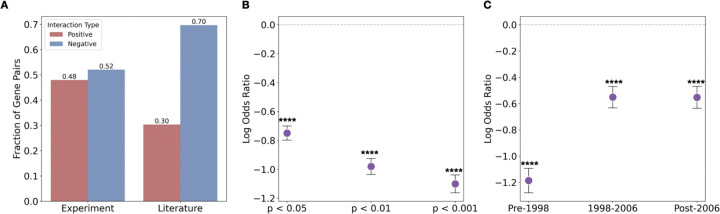
Positive interactions are underrepresented in the literature. **(A)** Proportion of gen epairs with positive (red) and negative (blue) interactions (computed using interactions with p<0.05) among experimentally identified pairs (left) versus those co-studied in the literature (right). **(B)** Log Odds ratio (literature vs. experiment) of positive to negative interactions at varying p-value thresholds; values below zero indicate depletion of positives. **(C)** Log odds ratio over three publication periods shows consitent underrepresentation of positive interactions. All error bars represent standard errors. Significance levels are indicated as follows: NS, *P* ≥ 0.05; *P < 0.05; **P < 0.01, ***P < 0.001; ****P<0.0001.

Because gene-pairs exhibiting positive interactions tend to be associated with higher impact, we wondered if positively interacting gene pairs are also enriched in literature. We compared the fraction of positive and negative gene pairs in the literature versus those identified through unbiased experimental screening. To our surprise, positively interacting gene pairs are significantly underrepresented in the literature compared to their prevalence in unbiased experimental screens (p<0.0001, Fisher’s exact test, Figure 2A). For instance, while approximately 48% of measured gene pairs exhibit positive interactions, among co-studied gene pairs that are involved in significant genetic interaction, only 30% are positive interactions. This depletion suggests a systematic bias against studying positive interactions. This underrepresentation is further highlighted by Figure 2B, which plots the log odds ratio of positive to negative interactions in the literature compared to unbiased experimental screens (all p<0.0001, Fisher’s exact test). A negative log odds ratio indicates a depletion of positive interaction pairs in the literature. Notably, as we consider increasingly stringent p-value cutoffs to define significant genetic interactions, this log odds ratio becomes more negative, signifying that the underrepresentation of positive interaction pairs in the literature is more severe for highly significant genetic interactions. This underrepresentation has also persisted over all time periods examined (all p<0.0001, Fisher’s exact test, Figure 2C), where each time period contains approximately one-third of the total literature in our sample (with unequal intervals reflecting uneven publication rates), indicating a consistent bias in research focus.These findings demonstrate that despite their scientific importance, positive genetic interactions are vastly underexplored in the scientific literature.

## Discussion

Our research reveals a significant, yet overlooked, phenomenon in genetic studies: positive genetic interactions, despite being linked to higher-impact publications, are consistently understudied. This observation directly challenges the conventional wisdom regarding which genetic interactions are most informative. It also resonates with established ideas in the sociology of science, which highlight the profound potential of unexpected discoveries.

We acknowledge a few limitations in our study. First, our reliance on literature curated by SGD might inadvertently favor highly cited papers. However, this potential bias should apply equally to both positive and negative genetic interactions, and thus not undermine our primary conclusions. Second, while citations are not a perfect metric of scientific impact due to social influences like prestige^10^, there is no evident reason for such bias to disproportionately affect studies on positive versus negative interactions, making its impact on our results improbable. Finally, one could argue our findings are subject to survivorship bias, where only exceptionally strong positive interactions are studied, artificially inflating their perceived impact. Our data, however, indicate the opposite: negative interactions reported in the literature exhibit greater absolute strength (0.4452, 95% CI: [0.4295, 0.4609]) compared to positive ones (0.1196, 95% CI: [0.1134, 0.1257]). This suggests that if positive interactions were studied at comparable strengths to negative interactions, their citation advantage would likely be even more pronounced.

More broadly, our study highlights the value of integrating sociological insights and “science-of-science”^14^ methodology to critically assess research strategies in biomedical science. Using bibliometric analyses, it becomes possible to empirically evaluate widely used research heuristics, such as the hypothesis that assays enhancing biological function yield more valuable insights than those that disrupt it ^15^. We contend that developing this kind of meta-knowledge will be immensely beneficial to the biomedical community, fostering a more evidence-based approach to research design.

## Materials and Methods

### Data

Data for this study were drawn from three primary sources. Genetic interaction data were obtained from the Costanzo et al. (2016) large-scale screen (https://boonelab.ccbr.utoronto.ca/supplement/costanzo2016/), focusing on 1,331,306 significant non-essential gene pairs (p<0.05). This selection prioritized interaction scores derived from knockout experiments, considered the gold standard, and avoided trivial positive interactions often associated with essential genes^16^. Gene literature data were acquired from the Saccharomyces Genome Database (SGD) gene literature database (http://sgd-archive.yeastgenome.org/curation/literature/, gene_literature.tab file). This systematically curated database links 418,111 papers to their associated yeast genes. For our analysis, we first counted the number of genes associated with each paper, then retained only those papers linked to precisely two genes. This specific focus aligns with our definition of genetic interaction, which is inherently between two genes, and simplifies our analysis by allowing us to clearly attribute scientific impact to specific gene pairs rather than needing to deal with the complexities of multi-gene relationships. Bibliometric data were sourced from the iCite database (https://nih.figshare.com/authors/iCite/7026758), which provides citation information for all PubMed literature. We matched the SGD gene-pair literature to iCite using PubMed IDs (PMIDs) and filtered for ‘research articles’ as defined by iCite, resulting in 1,981 papers with citation data associated with gene pairs involved in significant genetic interactions.

### Scientific Impact Metrics

Scientific impact was assessed using two different year-normalized metrics. The primary metric was year-specific percentile citation rank, defined as the percentile of a paper’s citation count within all gene literature articles published in the same year that could be linked to iCite. This method provides fair comparisons across publication years, accounting for citation accumulation over time. The alternative metric, year-normalized citation impact, was calculated by dividing a paper’s citation count by the average citation count for papers published in the same year.

### Novelty Mediation Analysis

To assess whether the higher impact of positive genetic interactions is driven by their novelty—specifically, their tendency to connect functionally distant genes—we computed GO-term similarity for each gene pair using GOATOOLS^17^. Annotations from the Saccharomyces Genome Database GAF file were propagated to all parent terms, and for each pair we averaged all pairwise similarities under two complementary metrics: Wang’s graph-based measure of structural overlap in the GO hierarchy, and Lin’s information-content measure emphasizing term specificity. To isolate novelty’s effect, we paired every positive interaction with a negative interaction by calculating the Mahalanobis distance between their (Wang, Lin) similarity vectors and solving the resulting assignment problem to minimize overall distance, yielding one-to-one matched sets without discarding any positives. We also created a size-matched random control by sampling negatives equal in number to the positives. These matched and random datasets were then used for testing the hypothesis that higher impact of positive genetic interactions is driven by their novelty.

## Data Availability

All codes and data that are necessary to reproduce the analysis in the manuscript will be published as a github repository upon publication.
